# Mammographic Criteria for Determining the Diagnostic Accuracy of Microcalcifications in the Detection of Malignant Breast Lesions

**DOI:** 10.7759/cureus.5919

**Published:** 2019-10-16

**Authors:** Qurat Hadi, Imrana Masroor, Zainab Hussain

**Affiliations:** 1 Radiology, Dow Institute of Radiology (OJHA Campus), Dow University of Health Sciences, Karachi, PAK; 2 Radiology, Aga Khan University Hospital, Karachi, PAK

**Keywords:** birads, breast cancer, pleomorphic microcalcifications, fine branching microcalcifications

## Abstract

Background

Breast cancer is a progressive disease, with conditions secondary to primary breast cancer being among the more common causes of malignancy-related deaths in women. Early diagnosis can halt disease progression and significantly improve patient's survival. Microcalcifications detected on mammograms may be an indicator of breast cancer. This study assessed the diagnostic accuracy of microcalcifications seen on mammograms for the detection of malignant breast disease when compared with histopathology.

Materials and methods

This study enrolled 144 women referred to the Radiology Department of Aga Khan University Hospital in Karachi, Pakistan, for mammograms and who were found to have suspicious microcalcifications, for which they underwent subsequent biopsy with histopathology over one year. The accuracy of microcalcifications, along with their sensitivity, specificity, negative predictive value (NPV), and positive predictive value (PPV), were assessed relative to histopathology results.

Results

Compared with histopathology results, microcalcifications had a sensitivity of 88%, and specificity of 62.8%, a PPV of 55.7%, and an NPV of 90.8%. The overall accuracy of microcalcifications was 71.5%.

Conclusions

The presence of microcalcifications on mammograms may predict breast malignancy. Studies with larger numbers of patients are required to determine whether microcalcifications have higher specificity and PPV relative to breast histopathology.

## Introduction

Conditions secondary to primary breast cancer are among the most common causes of malignancy-related deaths in women [1]. Worldwide, 21% of patients newly diagnosed with cancer are found to have conditions secondary to breast cancer, with average lifetime risks of about 6.2% in developed countries and 2.2% in developing countries [2]. A study in the Netherlands estimated that the prevalence of breast cancer is 6.3%, consistent with the results of studies reporting that more than 50% of breast cancer patients worldwide live in developing countries, where resources to fight this disease are insufficient [3].

The burden of breast cancer disease is higher in Pakistan than in other Asian countries [4-5]. The Karachi Cancer Registry has reported that breast cancer is the most frequent cancer (34.6%) among women in Pakistan, a country with one of the highest rates of breast cancer incidence worldwide, with approximately one in every nine women having a lifetime risk of breast cancer [4]. Moreover, breast cancer tends to present at a more advanced stage in Pakistani women than in women in western countries [5].

Studies in western countries have shown that screening mammography reduces breast cancer mortality by 40% to 45% [6]. Mammography is therefore recommended for the early detection of breast cancer, with a significant percentage of women aged more than 40 years in the United States enrolling in these screening programs [7].

Despite the progressive nature of the disease, its progress can be halted by early diagnosis and timely management [8]. High-resolution mammography with a low dose film screen is the standard technique for early identification of breast cancers, with about 25% to 43% of non-palpable cancers identified on mammography by the presence of calcifications [1]. The presence of microcalcifications on mammography has resulted in the detection of 62% to 98% of ductal carcinomas in situ (DCIS) [9]. Mammography was found to have a sensitivity of 95.2% and a specificity of 41.4% in detecting microcalcifications [10]. The American College of Radiology (ACR) standardized reporting system, called the Breast Imaging Reporting and Data System (BI-RADS) [11] has classified microcalcifications associated with breast cancer as pleomorphic or heterogeneous and as fine and/or branching (casting) calcifications.

A study in Japan assessing the positive predictive value (PPV) of different categories of microcalcifications according to BI-RADS found that 92% of linear and 67% of pleomorphic microcalcifications were malignant [12], and an evaluation of the PPV according to the BI-RADS classification reported that interobserver agreement was fair [13]. Additional studies are needed to improve the accuracy and standardization of mammographic findings of microcalcifications. To our knowledge, no such study has been performed to date in Pakistan. The present study, therefore, evaluated the associations between different types of microcalcifications and breast malignancy.

## Materials and methods

This prospective, cross-sectional analytical study involved patients referred to the Department of Radiology of Aga Khan University Hospital in Karachi, Pakistan. The sample size was estimated based on World Health Organization software determinations of sample size in health studies. Microcalcifications have been reported to have a sensitivity of 95.2% and a specificity of 41.4% in detecting breast cancers, and 34.6% of Pakistani women with cancer have been reported to have breast cancer. Thus, based on a 95% confidence interval and a margin of error of 10%, 144 patients are required to evaluate the diagnostic accuracy of microcalcifications on mammography in identifying malignant breast lesions. Non-probability, purposive sampling was used.

The present study enrolled all women referred to the Radiology Department of Aga Khan University Hospital for mammography, who were found to have suspicious microcalcifications (pleomorphic and fine branching) and underwent subsequent biopsy with histopathologic examination. Patients with a previous history of breast cancer were excluded, as were patients who underwent evaluation at another hospital with non-availability of histopathology results.

Written informed consent was obtained from all included patients. Mammography was performed on a MAMMOMAT NOVA 3000 Siemens (Amber Diagnostics, Inc., Orlando, FL) at 26--30 kVp, with images acquired in mediolateral, oblique, and craniocaudal projections. The mammograms were analyzed by an experienced consultant radiologist with at least five years of experience, and the histopathology results were analyzed by an experienced pathologist with at least five years of experience. True positives were defined as mammograms showing microcalcifications associated with malignancy (pleomorphic and fine branching type), with breast cancer subsequently confirmed on histopathology examinations. True negatives were defined as mammograms showing benign-appearing microcalcifications, with non-malignancy subsequently confirmed by histopathology.

Mammogram findings were compared with histopathology results, regarded as the gold standard. All statistical analyses were performed using SPSS for Windows, Version 16.0 (SPSS Inc., Chicago). The sensitivity, specificity, PPV, negative predictive value (NPV), and diagnostic accuracy of microcalcifications in the detection of breast cancer were calculated relative to histopathology. Age was reported as mean and standard deviation (SD), whereas pleomorphic, fine branching, and non-malignant microcalcifications; family history of breast cancer, nulliparity and use of hormone replacement therapy (HRT) were reported as frequencies and percentages. Patients were stratified by age, family history of breast malignancy, use of HRT and nulliparity, and the effects of these variables on outcomes were calculated.

## Results

The present study enrolled all women who were referred to our department for suspicious breast microcalcifications. Histopathology showed that 50 (35%) of these patients had biopsy-proven breast carcinoma. Mammography correctly identified malignancy in 44 (88%) of these 50 patients, but incorrectly identified benign lesions in six (12%) patients (Table 1). Histopathologic examination showed no evidence of malignancy in the remaining 94 (65%) patients; however, mammography incorrectly identified 35 (37.2 %) of these patients as suspicious for malignancy. The accuracy of mammography in evaluating microcalcifications was 71.5%.

**Table 1:** Statistical evaluation of the ability of microcalcifications to predict breast cancer Abbreviations: NPV, negative predictive value; PPV, positive predictive value

Total patients	144
True positive	44
True negative	59
False positive	35
False negative	6
Sensitivity	88%
Specificity	62.8%
Accuracy	71.5%
NPV	90.8%
PPV	55.7%

Pleomorphic and fine branching microcalcifications (malignant) were present in 73 (50.7%), and six (4.2%) patients, respectively. Other (benign) microcalcifications were observed in 65 (45.1%) patients (Table 2).

**Table 2:** Demographic and clinical characteristics of patients included in this study Abbreviations: HRT, hormone replacement therapy; SD, standard deviation.

	N	Percent
Age, year, mean ± SD	144	55.31 ± 9.79
Positive family history	51	35.4
Nulliparity	15	10.4
Use of HRT	19	13.2
Pleomorphic microcalcifications	73	50.7
Fine branching microcalcifications	6	4.2
Other microcalcifications	65	45.1
Histopathologic evidence of breast cancer	50	35

The enrolled patients ranged in age from 31 to 82 years, with a mean ± SD age of 55.31 ± 9.79 years. The largest age group consisted of patients aged 51-60 years, accounting for 56 (38.9%) of the 144 patients. The age group with the highest percentage of patients having breast carcinoma was aged 31-40 years (5/9, 56%).

Of the 144 patients, 51 (35.4%) had a family history of breast cancer, 15 (10.4%) were nulliparous, and 19 (13.2%) had taken HRT.

## Discussion

Breast cancer is one of the most common causes of cancer deaths among women [1]. Early recognition and effective treatment of women diagnosed with breast cancer are keys to reducing mortality rates [8]. Although breast self-examination and regular clinical examination have enhanced early detection of breast cancer, imaging is of paramount importance due to the occult nature of this disease.

Mammography has been found to be an effective screening test for the timely identification of breast cancer. Owing to the presence of microcalcifications, mammography has been found to detect 25%-43% of non-palpable cancers [1], including 62%-98% of DCISs [9]. The association between microcalcifications and breast malignancy is not specific; however, microcalcifications are also present in women with benign breast diseases, including fibrocystic changes. Many radiological criteria have been proposed to differentiate malignant from benign calcifications [13], including the ACR formulated BI-RADS, most recently revised in 2013 [11].

The present study found that the overall diagnostic accuracy of mammography in detecting microcalcifications associated with breast cancer was 71.5%, with a sensitivity of 88%, a specificity of 62.8%, a PPV of 55.7%, and an NPV of 90.8%. These findings were in agreement with those of a previous study, which reported that mammography had a sensitivity of 95.2% and a specificity of 41.4% in detecting malignant microcalcifications [10].

**Figure 1:**
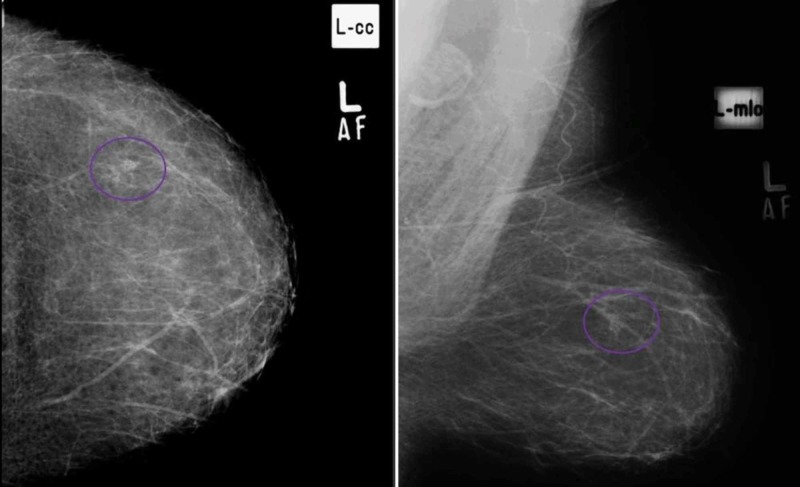
(A) Craniocaudal and (B) mediolateral oblique views in a patient with cluster of pleomorphic microcalcifications in upper outer quadrant of left breast, proven to be breast carcinoma on histopathology. (True Positive)

Of the 144 patients included in this study, 73 (50.7%) had pleomorphic calcifications on mammography, with 39 of the latter (53.4%) being histopathologically positive for breast cancer. Thus, the diagnostic accuracy of mammography in detecting breast carcinoma in patients with pleomorphic calcifications was calculated to be 71.0%. In contrast, only six patients (4.2%) had fine branching microcalcifications on mammography, with five (83.3%) of these patients being histopathologically diagnosed with breast malignancy. The diagnostic accuracy of mammography in detecting breast carcinoma in patients with fine branching microcalcifications was, therefore, 91.5% (Figure 1A, 1B).

In comparison, a study from Japan found that pleomorphic microcalcifications and fine branching microcalcifications on mammography had PPVs of 67% and 92%, respectively, for detection of malignancy [12]. Another study found that two readers had PPVs of 17% and 25%, respectively, for category 4, and 68% and 44%, respectively, for Category 5 breast microcalcifications according to the BI-RADS classification [13]. Similarly, fine pleomorphic lesions on mammography had a PPV for breast malignancy of 28% [14], and linear calcifications had a PPV of 81% [15]. The results of the present study are consistent with these previous findings.

**Figure 2:**
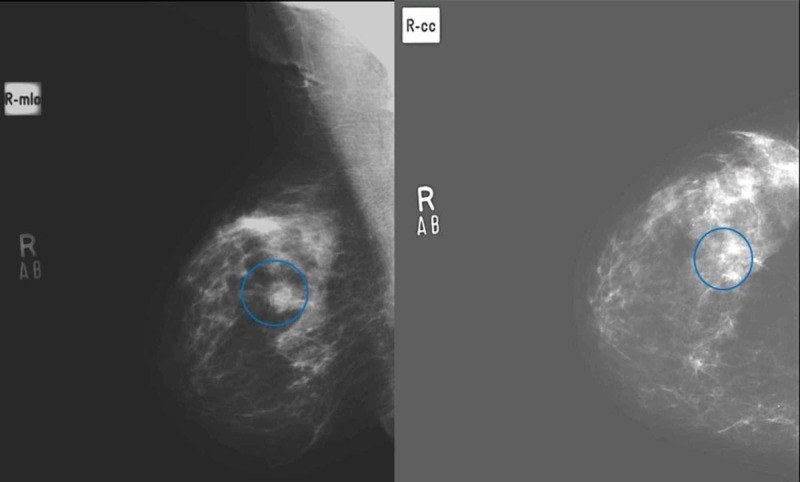
(A) Medio lateral oblique and (B) craniocaudal views in a patient with scattered punctate microcalcifications in both breasts, histopathology revealed fibrocystic changes with intraductal papilloma on histopathology. (True negative)

**Figure 3:**
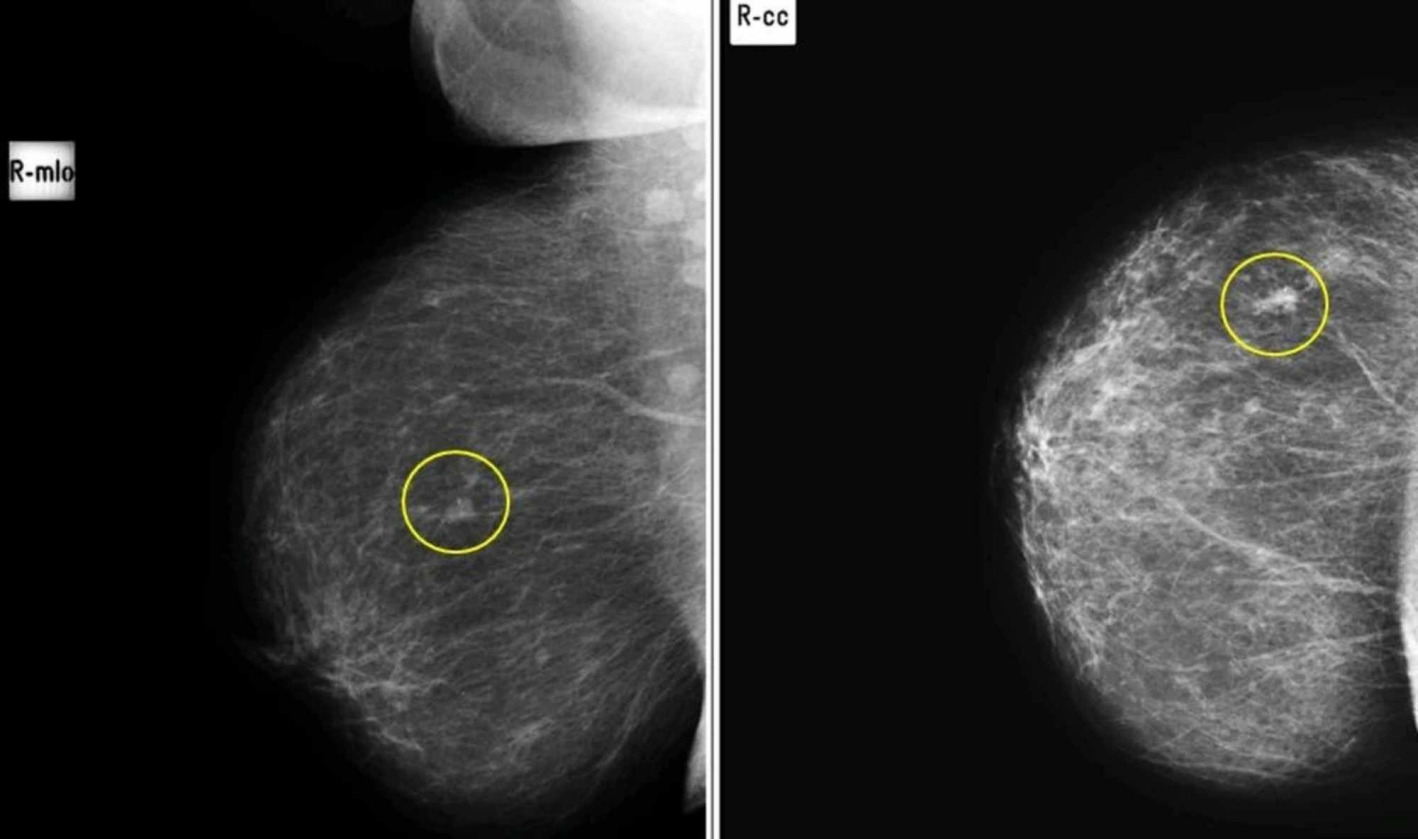
Mediolateral (A) and craniocaudal (B) views in a patient with soft tissue nodule with benign specks of microcalcifications in upper outer quadrant of right breast, proven to be breast carcinoma on histopathology. (False Negative)

Mammography detected other (benign) microcalcifications in 65 (45.1%) of our 144 patients, with 59 (90.8%) diagnosed as benign and six (9.2%) diagnosed as malignant on histopathology (Figure 2A, 2B). The patients with false-negative results showed punctuate/amorphous calcifications (Figure 3A, 3B). As in our study, the BI-RADS classifies punctate calcifications as benign. One study found that, of 141 lesions, three DCISs had punctate, coarse, and coarsening microcalcifications, respectively, on mammography [16]. The higher rate of false negatives in our study may have been due to our classification of amorphous and coarse heterogeneous calcifications as benign. In contrast, the BI-RADS categorizes these lesions as intermediate calcifications, with studies showed that these microcalcifications are associated with an increased risk of malignancy [12,17]. In comparison, amorphous and coarse heterogeneous calcifications were reported to have PPVs of 20% each [14], and 26% of breast carcinomas were found to have amorphous microcalcifications [15]. 

**Figure 4:**
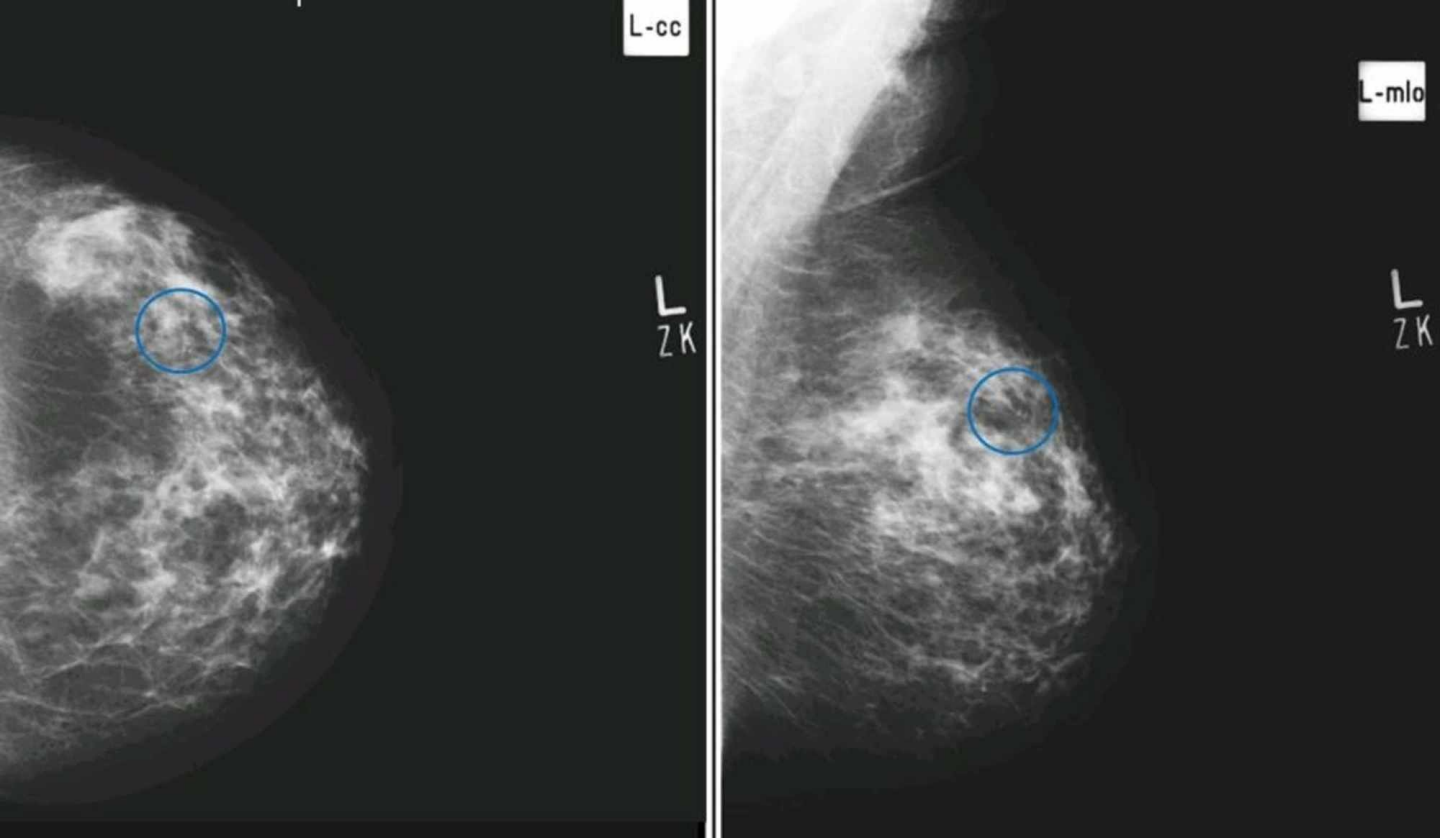
Craniocaudal (A) and mediolateral oblique (B) views in a patient showing early pleomorphism in the upper outer quadrant of left breast, however, histopathology revealed benign breast tissue with fibroadenomatoid change and stromal calcifications. (False Positive)

In our study, microcalcifications in 35 patients, including 34 pleomorphic and one fine linear branching microcalcifications, were interpreted on mammography as positive results but found to be negative on histopathology (Figure 4A, 4B). Histopathologic examination of biopsies of these 35 lesions showed that 25 of these patients had foci consistent with fibrocystic changes/fibroadenomatosis/sclerosing adenosis, eight had duct ectasia/cysts, seven had columnar cell changes with nuclear atypia in one patient, six had apocrine metaplasia, five had benign breast tissue, two had papillomas, and one each had a fibroadenoma and fat necrosis. Some of these lesions can present as pleomorphic calcifications [18-23].

Fibrocystic breast disease is a common condition among premenopausal women, resulting from different combinations of basic histologic lesions, including epithelial proliferations, lactiferous cysts, stromal fibrosis, sclerosing adenosis, and apocrine metaplasia [18]. Fibrocystic breast disease may be associated with other benign breast disorders, such as atypical lobular hyperplasia, and may manifest on mammography as clusters of microcalcifications, simulating low-grade DCIS [19].

Sclerosing adenosis, a form of fibrocystic change, frequently mimics breast carcinoma. Mammographic findings of sclerosing adenosis include microcalcifications; circumscribed, ill-defined, and spiculated masses; focal asymmetry; and focal architectural distortion [20]. Benign papillomas are another common type of breast lesion that can mimic carcinoma. Mammographically, benign papillomas present as single or multiple circumscribed or irregular masses, with or without microcalcifications [21]. Occasionally, fibroadenomas may contain small punctate, dystrophic, or pleomorphic calcifications [22]. Fat necrosis can present as various types of mammographic abnormalities, including clustered pleomorphic microcalcifications [23].

The mean age of the patients in our study was 55.31 years, with patients ranging in age from 31 to 82 years, and 56 patients aged 51 to 60 years. Patients with suspicious microcalcifications later confirmed histopathologically as being breast cancer ranging in age from 31 to 74 years. Of the various subgroups by age, patients aged 31 to 40 years contained the highest percentage with breast carcinoma (5/9, 56%), a finding that may be due to improved detection of breast carcinoma in younger women. A study assessing the survival of patients in Lahore, Pakistan, with locally advanced breast cancer found that the median age of breast cancer patients was 45 years, with the majority being pre-menopausal and having the receptor-negative disease [24]. 

Family history has been reported to be a significant risk factor for breast cancer development, especially in younger women. Of the 50 women with histologically proven breast cancer in our study, five (10%) had a positive family history. In comparison, a population-based survey reported that positive family history for various types of cancer had a sensitivity of 61.1%, a specificity of 95%, an NPV of 61.3% and a PPV of 95% for the same type of cancer [25].

Nulliparity is a documented risk factor for breast malignancy, particularly when compared with parity in young women. Nulliparous women have a higher risk (20%-40%) of developing post-menopausal breast malignancy than women who had their first child before the age of 25 years [26]. Of the 50 nulliparous women in our study, six (12%) had breast cancer. Primary data from four National Cancer Institute prospective studies found that nulliparous women had a 38% higher risk of breast cancer than women who had their first child before the age of 25 years, with the overall risk of breast cancer being 11% higher among nulliparous than parous women [27].

HRT may also be associated with an increased frequency of breast cancer in postmenopausal women based on evidence from three studies, the Collaborative Reanalysis (CR), the Women's Health Initiative (WHI), and the Million Women Study (MWS). Of the 50 women in our study using HRT, five (10%) were found to have breast cancer. A meta-analysis showed that women taking HRT, particularly combinations of estrogen and progesterone, were at higher risk of breast cancer than women taking estrogen-only regimens [28]. In Pakistan, however, HRT is not a frequent choice and is not being recommended by physicians.

Breast mammography is regarded as the best tool for assessing microcalcifications. Thus, other breast imaging tools, such as ultrasound and magnetic resonance imaging, cannot replace mammography in the early detection of breast malignancy [12]. Our results confirm that mammographic determinations of microcalcifications are a sufficiently sensitive and accurate tool for detecting breast cancer. However, multicenter studies including larger numbers of patients are needed to assess the specificity and PPV of this method.

The ACR BI-RADS utilizes many terms to describe the morphology of microcalcifications. Each term is used to rank lesions into categories that can be used to estimate their malignant potential. These categories include benign, intermediate concern, and a higher probability of malignancy [11]. Although the BI-RADS does not classify descriptions of microcalcification distribution into risk grades, these descriptions, especially of linear and segmental patterns, can help in estimating the possibility of malignancy [12,15,17].

The outcomes of this study and their confirmation of previous results suggest that suspicious microcalcifications, including pleomorphic and fine branching calcifications, should be biopsied prior to any operative intervention.

This study had several limitations. First, this study included a relatively small number of patients presenting at a single center; therefore, the results cannot be generalized. Second, this study, unlike previous studies, did not assess the PPVs of morphology and distribution simultaneously. Third, amorphous and coarse heterogeneous calcifications were considered benign rather than intermediate, as in the BI-RADS classification.

## Conclusions

These findings confirmed that mammographic determination of microcalcifications is an adequately sensitive and accurate tool for the detection of breast cancer. This study also showed that morphological descriptions of microcalcifications and BI-RADS categories are helpful in predicting the risk of malignancy of suspicious microcalcifications. However, multicenter studies with larger numbers of patients are required to further evaluate the specificity and PPV of morphology and distribution simultaneously.
